# Open repair of a giant hepatic artery aneurysm

**DOI:** 10.1093/jscr/rjaf799

**Published:** 2025-10-07

**Authors:** Arunan Mahendravarman, Christopher Seng Hong Lim, Jaswinder Singh Samra

**Affiliations:** Department of Upper Gastrointestinal Surgery, Royal North Shore Hospital, Reserve Road, St Leonards, New South Wales 2065, Australia; Department of Upper Gastrointestinal Surgery, Royal North Shore Hospital, Reserve Road, St Leonards, New South Wales 2065, Australia; Department of Upper Gastrointestinal Surgery, Royal North Shore Hospital, Reserve Road, St Leonards, New South Wales 2065, Australia

**Keywords:** hepatic artery, aneurysm, follow up, surgery

## Abstract

Hepatic artery aneurysms (HAAs) are rare vascular lesions with significant risk of rupture and high mortality if untreated. We report a 59-year-old male who re-presented with severe chest and abdominal pain 10 years after failed embolization of a known HAA. Imaging revealed a massive lesion with extensive local mass effect, ultimately requiring complex multivisceral resection and vascular reconstruction to achieve definitive management.

## Introduction

We present a rare case of a giant hepatic artery aneurysm (HAA) in a 59-year-old male who re-presented with acute chest and abdominal pain, 10 years after failed coil and glue embolization and loss to follow-up. Imaging revealed a massive right upper quadrant aneurysm measuring 23 cm, causing significant compression of surrounding structures. Definitive management required complex open resection involving pancreaticoduodectomy, splenectomy, distal gastrectomy to gain access, resection and reconstruction of portal vein, and superior mesenteric vein with hepatic artery re-anastomosis. Histopathology confirmed mixed aneurysm and pseudoaneurysm. HAAs are rare, comprising <0.5% of arterial aneurysms, and giant HAAs (>5 cm) are exceedingly uncommon, with this case representing the largest described to date. This case underscores the critical importance of surveillance and timely intervention for HAAs to prevent catastrophic complications, such as rupture, and highlights the technical challenges posed by giant aneurysms with extensive visceral involvement.

## Case report

We report a case of a 23 cm giant hepatic artery aneurysm (HAA), the largest reported in the literature to date. A 59-year-old male presented to the hospital with acute chest and abdominal pain. On examination, there was a large non-pulsatile mass in the right upper abdomen without features of peritonism. His biochemistry was notable for global liver function enzyme derangement (bilirubin 30 umol/L, alkaline phosphatase 1021 unit/L, gamma-glutamyl transferase 2648 unit/L, alanine aminotransferase 210 unit/L, and aspartate aminotransferase 125 unit/L).

**Figure 1:**
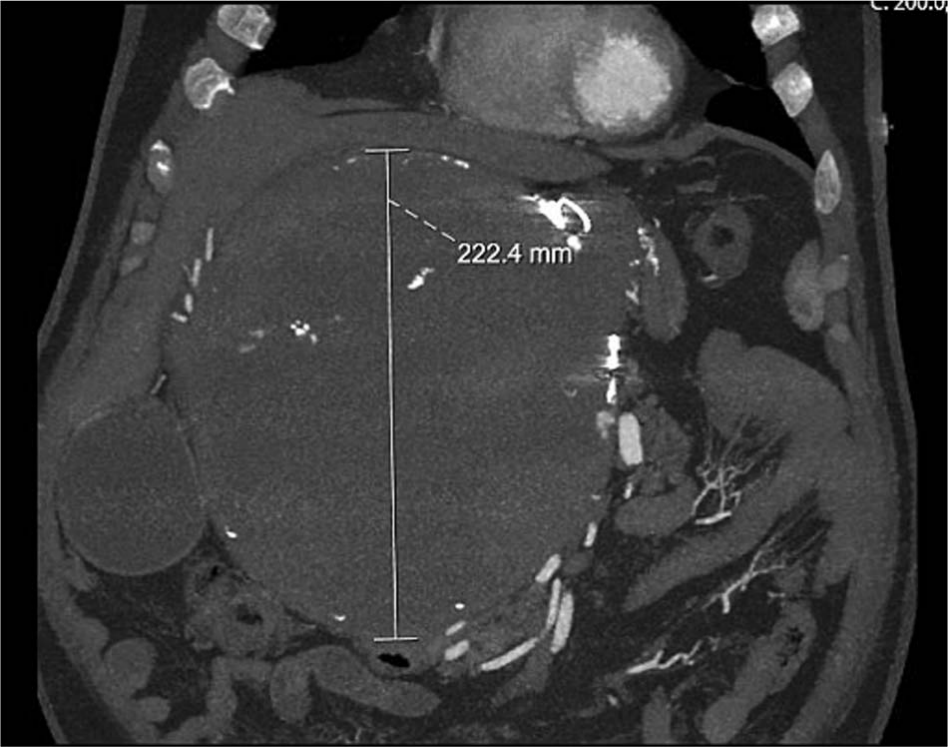
Coronal CT image showing large HAA.

A computed tomography (CT) aortogram performed to exclude an aortic dissection detected a large right upper quadrant lesion measuring 230 × 178 × 224 mm (Fig. 1). A targeted multiphase CT abdomen and pelvis again revealed a large mass with heterogeneous internal attenuation and high peripheral attenuation. A small feeding vessel was identified inferiorly adjacent to its junction with the common hepatic artery. Significant mass effect on surrounding structures was demonstrated with partial compression and displacement of the pancreas, spleen, duodenum, liver, aorta, and inferior vena cava.

On review of medical records, it was found that this lesion was previously diagnosed 10 years earlier measuring 7.5 × 7.1 cm. The patient underwent intervention for this lesion with coil and glue embolization but was unsuccessful. He was advised to follow-up with repeat imaging in 6 months but unfortunately failed to attend.

**Figure 2:**
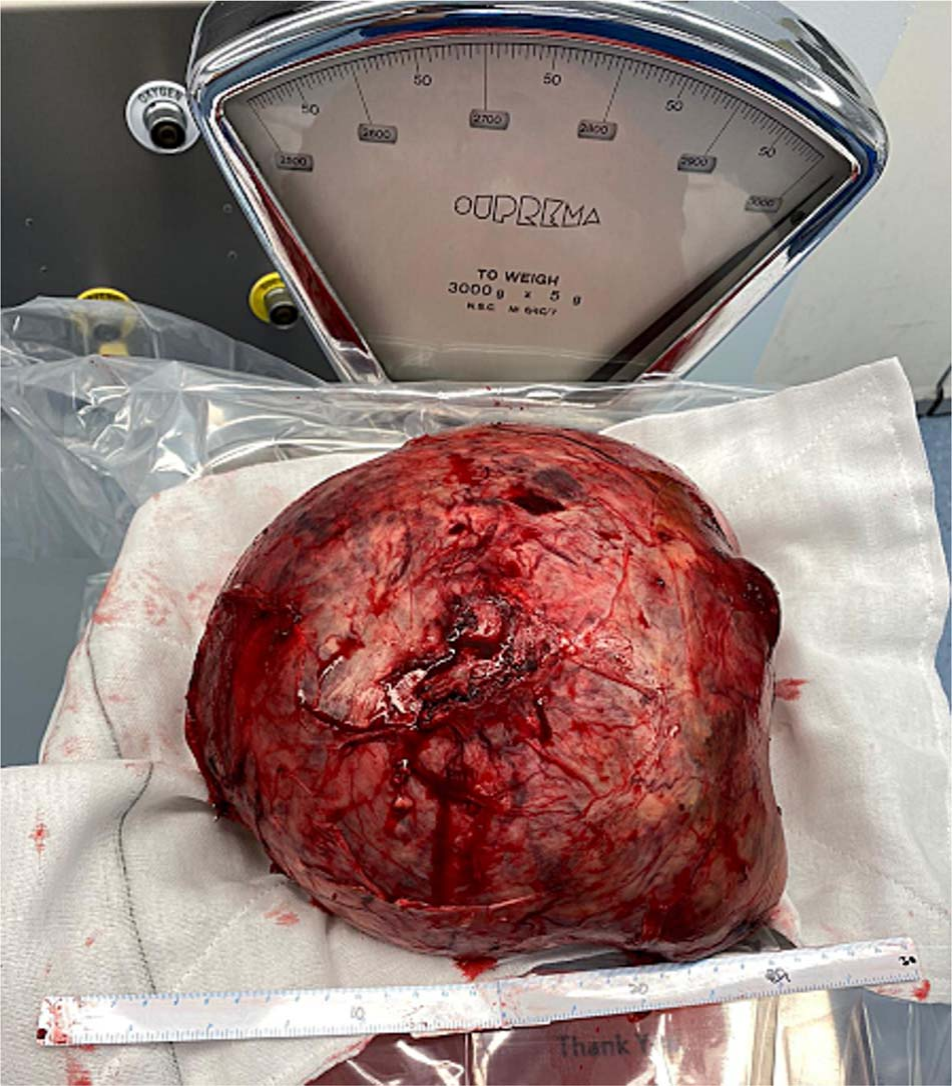
Large HAA post removal.

At representation, due to its immense size, an operative approach was adopted, which was led by a senior hepatopancreaticobiliary surgeon alongside two senior vascular surgeons. At laparotomy, a large aneurysm that was densely adherent to the posterior surface of the stomach and pancreas was found. A pancreatoduodenectomy was performed to facilitate exposure. The common hepatic artery proximal to the aneurysm was divided and oversewn. Next, the hepatic artery distal to the aneurysm just proximal to the bifurcation was divided and anastomosed to the gastroduodenal artery which was supplied from below via the inferior pancreaticoduodenal branches of the superior mesenteric artery. The portal vein was divided to free the aneurysm (Fig. 2) and was subsequently reconstructed with an end-to-end anastomosis. A completion pancreatectomy and splenectomy were performed at the index operation due to the high risk of complications to the vascular anastomoses in the event of a pancreatic fistula. Histopathology confirmed mixed aneurysm and pseudoaneurysm. The patient remained in hospital for a total of 24 days and is due to follow up with repeat multiphase CT angiograms for surveillance.

## Discussion

Hepatic artery aneurysms comprise <0.5% of all arterial aneurysms, and giant HAA (diameter > 5 cm) are exceedingly rare [1]. HAA are commonly asymptomatic and diagnosed incidentally on imaging. They are associated with rupture rates of 14%–80% with a risk of death following rupture of up to 40% [2, 3]. Therefore, follow-up of HAA following diagnosis is important to allow early detection of progression and to guide management.

HAA are characterized as either extrahepatic (80%) or intrahepatic (20%). They are twice as common in men and the mean age at diagnosis is in the sixth decade. While most HAA are asymptomatic and incidentally diagnosed, CT angiography is the modality of choice to distinguish between vascular and soft tissue aetiologies.

The optimal management of asymptomatic HAA is debatable due to a lack of knowledge about risk factors for rupture. In general, annual follow-up with CT angiography is recommended for asymptomatic HAA < 2 cm [4]. Management options for larger HAA include endovascular and surgical interventions or surveillance and largely depend on individual patient factors.

There have been several cases of giant HAA managed successfully by endovascular embolization; however, no cases utilized coil embolization alone [5–7]. Coil embolization of giant HAA is associated with hepatic ischaemia, persistent perfusion, or secondary recanalization of the aneurysm likely due to their large size [1, 8]. As in our case, despite coil and glue embolization, the HAA still showed residual filling.

The largest HAA previously recorded in the literature measured 14 cm in diameter [9]. This was managed with open aneurysmectomy and bypass grafting.

We present the largest giant HAA in the literature, which was significantly involved with surrounding abdominal organs and vessels requiring extensive visceral resection and vascular reconstruction. This case highlights the importance of close follow-up of patients with HAA to allow early detection and intervention to reduce the risk of complications including rupture.
